# Savoring Daily Life: Views of Aging as a Moderator of Savoring and Positive Affect

**DOI:** 10.1093/geroni/igaf122.492

**Published:** 2025-12-31

**Authors:** Lydia Ong, Theresa Pauly, Anna Kornadt, Nancy Sin

**Affiliations:** The University of British Columbia, Vancouver, British Columbia, Canada; Simon Fraser University, Vancouver, British Columbia, Canada; University of Luxembourg, Esch-sur-Alzette, Grevenmacher, Luxembourg; University of British Columbia, Vancouver, British Columbia, Canada

## Abstract

Positive events are common in daily life (e.g., having a good conversation) and savoring strategies can be used to up-regulate positive emotions. Drawing from Socioemotional Selectivity Theory, individuals with relatively less positive views of aging (VoA) might attend more to positive and meaningful experiences in the present as a function of shifting time perspective. Therefore, we hypothesized that people with less positive VoA would derive more positive affect from savoring their positive experiences. This pre-registered study analyzed experience sampling data from an adult lifespan sample from Canada (n = 178 persons and 14 days, Mage=47) and an older adult sample from Switzerland (n = 108 persons and 7 days, Mage=73). VoA were measured through attitudes toward own aging and age stereotypes, while savoring and positive affect were assessed at the end of each day. Two-level multilevel models revealed that positive affect was higher on days with more-than-usual savoring (Canada sample: b = 0.16, 95% CI[0.03, 0.30], p=.019; Switzerland sample: b = 0.42, 95% CI[0.35, 0.49, p<.001). VoA moderated the link between savoring and positive affect in the Swiss sample only, such that individuals with less positive VoA had smaller upticks in positive affect associated with savoring (simple slope for -1 SD: b = 0.34, SE = 0.05, p<.001), compared to those with more positive VoA (simple slope for +1 SD: b = 0.49, SE = 0.05, p<.001). Findings suggest that older adults with more positive VoA may benefit more from savoring, but it remains unclear whether VoA hold the same importance for savoring among younger and middle-aged adults.

